# Alternate Nitrogen Amendments for Organic Fertilizers

**DOI:** 10.1100/tsw.2001.454

**Published:** 2001-12-19

**Authors:** M.K.C. Sridhar, G.O. Adeoye, O.O. AdeOluwa

**Affiliations:** Organo-Mineral Fertilizer Research and Development Group, Division of Environmental Health, College of Medicine, University of Ibadan, Nigeria

**Keywords:** nitrogen, compost, organic fertilizer, Nigeria, environment, Gliricidia

## Abstract

The use of compost or manure in agriculture as an organic source of nutrients is common in many tropical, developing countries like Nigeria. One of the drawbacks of such materials is their low nitrogen (N) content (=1% N). Farmers commonly use chemical N fertilizers such as urea, calcium ammonium nitrate (CAN), and NPK formulations to obtain better crop growth and yield. These chemical supplements may have a negative impact on the environment through nitrate leaching into water, leading to eutrophication of surface waters that can affect public health. Gliricidia sepium, a fast-growing, tropical, perennial hedge plant was tested as a source of N in organo-mineral fertilizer formulations. Average nutrient content of Gliricidia is 3.8% N, 0.32% P, 1.8% K, 0.8% Ca, and 0.2% Mg. Using a sand culture and Amaranthus caudatus as a test crop, it was shown that amending commercial composts with 30% Gliricidia prunings would benefit many small-scale farmers and control environmental pollution.

## INTRODUCTION

Before the advent of mineral fertilizers about 150 years ago, manure and composts were practically the only sources of nutrients for crops[1]. However, after independence and the discovery of crude oil in the 1960s, the use of mineral fertilizers displaced organic sources as people found them easy to handle and to apply on their farms. In 4 decades, it was recognized that several problems occurred with intensive use of inorganic fertilizers. Some of these problems were reliability of fertilizer supply, high cost, N volatilization losses, soil degradation through erosion, and frequent overapplication with consequent pollution of surface and groundwater resources. These problems have been encountered in many countries.

As a result, most countries began to put emphasis on the advantages of applying organic manure or organic fertilizers to replenish dwindling soil nutrients. Among the various organic materials in use are composted city refuse, green manure, decomposed animal wastes, and farmyard manure, which are usually found in abundant quantities in most communities. In Nigeria, millions of tons of city refuse are produced annually, which has the potential to be composted and used to improve soil fertility. *Gliricidia sepium*[2,3], a common hedge plant in the humid tropics, was found to be useful as a source of green manure. Compared to other shrubs, *Gliricidia* has a high decomposition rate and N release[4]. *Gliricidia* is also productive, yielding from 2 to 15 t/ha/year of dry matter[2]. In a study carried out by Handayanto et al.[5], it was observed that the N mineralization rate of low-grade prunings from *Peltophorum dasyrrachis* was significantly improved by mixing with *G. sepium*.

Many researchers have reported the superiority of combined inorganic and organic fertilizers (organo-mineral fertilizers) over their performance separately[6,7]. However, Cooke[8] and Peverly and Gates[9] showed that organic fertilizers could indeed perform better for some crops. The use of organic matter as fertilizer in developing countries has received much less attention from economists because the beneficial effects are not visible immediately[10]. Araji and Stodick[11] found that the cost associated with the handling and spreading of manure ranges between 20 to 30% of the cost of commercial fertilizer. This cost differential indicates that the use of green manure can be less costly than using urea or any chemical amendment in compost. Even though the use of organo-mineral fertilizers is widely accepted globally, the inorganic constituents may still pose environmental problems to some extent.

In order to minimize environmental impact, there is a need for local materials that can supplement the N and other minerals. One such alternative identified is *G. sepium*, which grows extensively as a wild hedge plant. This paper reports the assessment of *G. sepium* for its suitability as an amendment for organo-mineral fertilizers being prepared from market and slaughterhouse wastes.

## MATERIALS AND METHODS

### Fertilizer Sources

- Grade B organo-mineral fertilizer (OMF): Compost was obtained from Pace Setter Organic Fertilizer Plant, developed by the authors for the Oyo state government. The compost was made from market and slaughterhouse wastes from Ibadan, in southwestern Nigeria. The unamended compost was prepared using an aerobic windrow system and was devoid of toxic and heavy metals. This is referred to as Grade B OMF.
- Grade A OMF: The Grade B OMF was amended with urea and bone meal to enrich with extra N and P and is referred to as Grade A OMF.
- *Gliricidia* prunings were obtained from agricultural land by harvesting fresh leaves, drying to constant weight at a temperature of 80°C, and milling to pass through a 0.2-mm sieve.

### Fertilizer Treatments

Grade A OMF, Grade B OMF, and *Gliricidia* were used in the greenhouse experiments. With these materials, seven treatments were prepared (letter-coded as shown below) and applied as follows:

- Soil alone with no treatments (control) P
- 20% dried *Gliricidia* prunings + 80% Grade B OMF A
- 30% dried *Gliricidia* prunings + 70% Grade B OMF B
- 40% dried *Gliricidia* prunings + 60% Grade B OMF C
- 50% dried *Gliricidia* prunings + 50% Grade B OMF D
- 100% dried *Gliricidia* prunings alone M
- Grade B (unamended) OMF alone N
- Grade A OMF alone O

The soil used in the experiments was obtained from the University of Ibadan campus, which from the composition may be considered as the worst of tropical eroded soil. The soil was sandy (with about 90% sand) and slightly acidic (with pH 6.6). It had very low levels of mineral nutrients and exchangeable cations. This soil was deliberately chosen for the experiment with a view of establishing the efficacy of the available nutrients from the treatments under study (Table 1). The soil was sieved to pass 2 mm to remove stones and other unwanted constituents. In each pot, 2 kg soil was mixed with various amendments. *Amaranthus caudatus* was used as the test crop.

### Greenhouse Experiment

The greenhouse experiment was designed for evaluating the efficacy of the various treatments. Each treatment was applied at 5, 10, and 15 t/ha in 2-kg plastic pots in a randomized complete block design. Soil weight was 1.8 kg per pot. *A. caudatus* was planted twice successively, for main and residual effects. The ambient air temperature was about 28 to 30°C. *A. caudatus* was planted 1 week after the incorporation of the various treatments.

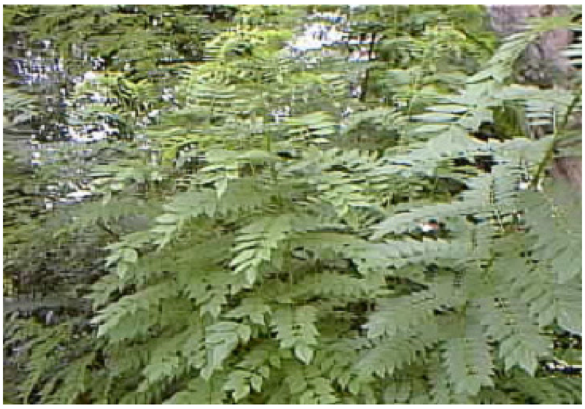

**FIGURE 1.** *G. sepium* in its natural habitat from a farm.

For finding the residual effect, fresh seeds were planted immediately after the first harvest, with no additional treatments. Water was applied daily to field capacity to maintain soil moisture content. If any weeds were seen, they were removed, ground, and put back into the same pots to account for nutrient loss. The following parameters were observed at 5 weeks after planting (WAP) and 6 WAP for residual effect: plant height, leaf number, stem girth, and whole-plant fresh and dry weight. Plant height was taken from the ground level to the dewlap of the last fully opened leaf. Stem girth was determined by passing a thread around a predetermined mark of 10 cm on the stem above the soil level. The harvested samples were oven dried to constant weight at 80°C and expressed as tons per hectare.

## Methods of Analyses

Six soil core samples (passed through a 2-mm sieve) were taken randomly for physical and chemical analysis. They were air-dried and used. Particle size distribution was determined by hydrometer method[12] using sodium hexametaphosphate as the dispersing agent. The coarse-sand fraction was separated from the fine sand using a 1-mm sieve.

Soil pH was determined by suspending the soil samples in distilled water (1:1) using glass electrode (EIL pH meter)[13]. Organic carbon was analyzed by the dichromate wet-oxidation method of Walkey-Black as described by Black[14], and the value of organic matter was obtained by multiplying the carbon level by 1.729. Total Kjeldahl N was determined by macro Kjeldahl method. Available P was determined by the Bray P (0.03 N $NH_4F$ + 0.025 N HCl) solution as described by Bray and Kurtz[15] and determined colorimetrically by the molybdate blue method.

Exchangeable cations were determined by leaching with 1N neutral $NH_4OAc$ according to the method of Tisdale[16]. K was determined by flame photometer as described by Sillanpaa[17]. Micronutrients (Mn, Fe, Cu, Zn) were extracted with 0.1 N EDTA and determined using atomic absorption spectrophotometer[18].

The results of the experiment were subjected to analysis of variance and the means compared using the Duncan Multiple Range test[19].

**TABLE 1**
**Physico-Chemical Properties of the Experimental Soil**

| Parameter | Value |
|---|---|
| pH value (in water) | 6.60 |
| Organic carbon, % | 1.50 |
| Total Kjeldahl N, g/kg | 1.3 |
| Available P, mg/kg | 7.50 |
| Exchangeable bases, cmol/kg | |
| K | 0.13 |
| Na | 7.50 |
| Mg | 0.18 |
| Ca | 0.14 |
| Exchangeable acidity, cmol/kg | 0.20 |
| CEC, cmol/kg | 4.28 |
| Base saturation, % | 95.50 |
| Extractable micronutrients, mg/kg | |
| Mn | 47.98 |
| Fe | 66.90 |
| Cu | 0.85 |
| Zn | 3.55 |
| Mechanical composition, g/kg | |
| Sand | 910.0 |
| Silt | 74.0 |
| Clay | 20.0 |

## RESULTS AND DISCUSSION

The Grade B OMF, which is the normal compost made from urban wastes, as expected was low in N and P (Table 2). The Grade A OMF was amended with urea and bone meal to increase N and P levels, respectively. Most farmers in Nigeria prefer a formula that can supply adequate N to meet requirements of certain high-N-demanding crops like maize and vegetables. P amendment is only needed for certain crops. In other African and Asian countries, the agricultural practice involves supplementing organic manure separately with inorganic fertilizers sometime during the growing period, usually after several weeks of growth. This practice is not common in Nigeria, where traditional fallowing methods or green manure are used when inorganic fertilizers are not available or cost effective.

*Gliricidia* has three to four times higher N content compared to normal compost (i.e., Grade B OMF). Therefore, the Grade B OMF was amended with ground dry leaves at various percentages from 0 to 100 along with appropriate controls (soil alone) to compare the efficacy in providing N to the test crop. Green amaranth is a widely used vegetable among the low- and middle-income traditional families. This test crop was monitored for the growth and dry-matter yield.

The highest *Amaranthus* yield at 5 WAP was 16.78 t/ha with the 30% *Gliricidia*-amended compost. Norman[20] reported a similar yield of 20 t/ha for amaranths grown on a sandy, infertile soil. If the soil is reasonably fertile, unlike the one used in this study, the yield can be substantial. Incorporation of *Gliricidia* improved the N levels of unamended compost (Table 2). While the 100% *Gliricidia*-prunings treatment proved to be most profitable, the 30% *Gliricidia* amendment seems to be ideal from the practical point of view. All the *Gliricidia*-amended composts indeed performed better than the urea-amended composts. The optimum application rate of amended composts was 15 t/ha both in the first and second crops (Table 3). The yields observed in the second crop may be due to residual nutrient availability. *Gliricidia* prunings gradually released nutrients over a period of 120 days[4]. Even the 40 and 30% *Gliricidia*-amended compost had significant residual effects compared to the urea-amended compost.

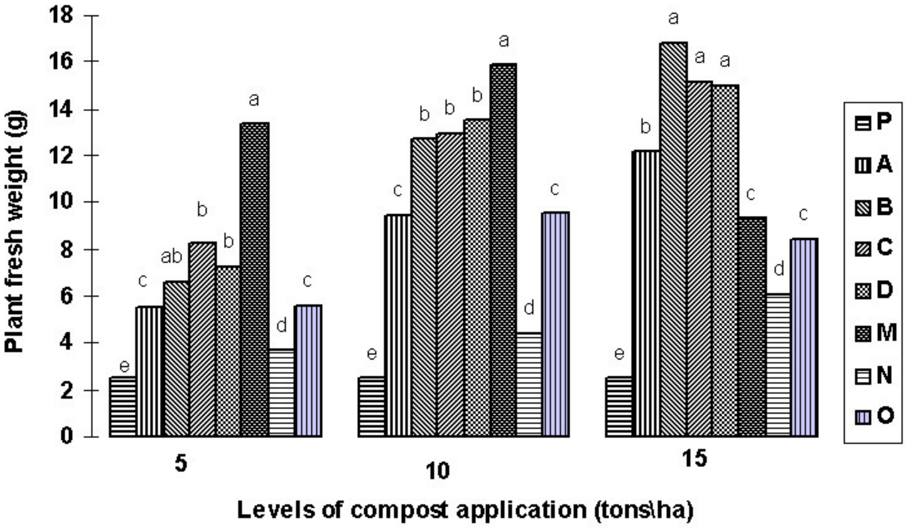

**FIGURE 2.** Effect of various fertilizers on fresh weight of *Amaranthus* at the end of 5 weeks after planting.

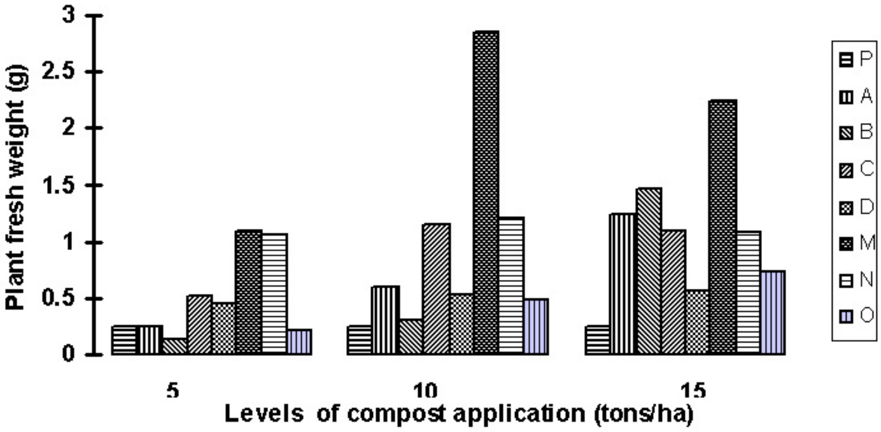

**FIGURE 3.** Residual effect of various fertilizers on fresh weight of *Amaranthus* at the end of 6 weeks after planting.

**TABLE 2**
**Nutrient Composition of Organo-Mineral Fertilizers and *G. sepium* Prunings**

| | Material Used | | |
|---|---|---|---|
| Composition | Grade A OMF | Grade B OMF | *G. sepium* |
| Total Kjeldahl N, % | 2.58 | 1.46 | 3.78 |
| Total phosphorus (P), % | 1.10 | 1.03 | 0.32 |
| Potassium (K), % | 0.68 | 0.60 | 1.83 |
| Calcium (Ca), % | 3.62 | 1.19 | 0.80 |
| Magnesium (Mg), % | 0.18 | 0.11 | 0.20 |
| Zinc (Zn), mg/kg | 276.0 | 290.0 | 31.00 |
| Copper (Cu), mg/kg | 25.0 | 22.00 | — |
| Manganese (Mn), mg/kg | 32.0 | 19.00 | — |
| Chromium (Cr), mg/kg | ND[a] | ND | — |
| Lead (Pb), mg/kg | ND | ND | — |

**TABLE 3**
**Estimated Income from *Amaranthus* Production Based on Best Level of Performance for Each Treatment**

| Treatments | | Yield (t/ha) Main Yield | Residual Effect | Total | Income/ha (NGN)[a] |
|---|---|---|---|---|---|
| Control | P | 2.5 | 0.25 | 2.75 | 27,500 |
| 20% *Gliricidia*-amended OMF | A | 12.19 | 1.24 | 13.43 | 134,300 |
| 30% *Gliricidia*-amended OMF | B | 16.78 | 1.46 | 18.34 | 183,400 |
| 40% *Gliricidia*-amended OMF | C | 15.16 | 1.09 | 16.25 | 162,500 |
| 50% *Gliricidia*-amended OMF | D | 15.03 | 0.56 | 15.59 | 155,900 |
| 100% *Gliricidia*-pruning | M | 15.88 | 2.85 | 18.73 | 187,300 |
| Unamended Grade B OMF | N | 6.04 | 1.07 | 7.11 | 71,100 |
| Urea-amended Grade A OMF | O | 9.58 | 0.48 | 10.06 | 100,600 |

[a] Currency equivalent: 1 USD = 114 NGN (Nigerian Nairas).

From an economic point of view (Table 3), the 100% *Gliricidia*-amended compost gave the best yield, and, in terms of currency, it amounted to about seven times higher than the control and almost double that of the urea-amended compost. However, it is not practicable to use the plant entirely, and the next best is 30%, which is very feasible when mixed with the composts prepared from wastes. When amended and applied at a rate of 10 t/ha, the economic returns are six times higher than the control. These results concur with Cooke[8] and Perverly and Gates[9], who reported that sole organic fertilizers performed better with some crops. Mafongoya et al.[21] studied the N-release patterns from seven leguminous trees and found that the lignin + polyphenol to N ratio could be used to screen leguminous tree leaves for their potential to release N. In another study by Lionel et al.[22] in Haiti, *G. sepium* was found to decompose faster as compared to seven other Hedgerow species. Leaves decomposed faster than stems. These studies further confirm the value of *Gliricidia* in compost amendments.

## CONCLUSIONS

The results of this study showed that *Gliricidia* prunings as an organic amendment for compost significantly improved growth and yield of *A. caudatus* compared to urea-amended compost. Cost and environmental advantages suggested that 100% *Gliricidia* prunings and their 30, 40, and 50% composition in compost could do better than urea-amended compost. Thus, the *Gliricidia* performed better in sandy soil than conventional urea used as a compost amendment. Based on results from the greenhouse study, the following conclusions may be drawn:

- Dry *Gliricidia* prunings are shown to be a good substitute for mineral fertilizers in enriching N levels of normal composts such as Grade B.
- 30% *Gliricidia* amendment of normal compost at an application rate of 15 t/ha optimized vegetable production, particularly when used in nutrient-depleted sandy soils.

| | | |
|---|---|---|
| Received: | July | 27, 2001 |
| Revised: | November | 12, 2001 |
| Accepted: | November | 15, 2001 |
| Published: | December | 19, 2001 |
